# Supplementary material for: A software platform for real-time and adaptive neuroscience experiments
Source: Nat Commun. 2025 Nov 11;16:9909. doi: 10.1038/s41467-025-64856-3 (PMC12606217; doi:10.1038/s41467-025-64856-3)
Supplement: Supplementary file 1 — Supplementary Information [file 41467_2025_64856_MOESM1_ESM.pdf]

Supplementary Information related to

improv a software platform for real-time and adaptive neuroscience experiments

Anne Draelos[1,2‡*], Matthew D. Loring[3], Maxim Nikitchenko[3], Chaichontat Sriworarat[1,2], Pranjal Gupta[2,4], Daniel Y. Sprague[1,2], Eftychios Pnevmatikakis[5], Andrea Giovannucci[6], Tyler Benster[7], Karl Deisseroth[7,8], John M. Pearson[1,2,3,4,9†], & Eva A. Naumann[3,4,10,11†*]

[1]Department of Biostatistics & Bioinformatics, Duke University School of Medicine, Durham, NC 27710, USA
[2]Center for Cognitive Neuroscience, Duke University, Durham, NC 27708, USA
[3]Department of Neurobiology, Duke University School of Medicine, Durham, NC 27710, USA
[4]Department of Psychology & Neuroscience, Duke University, Durham, NC 27708, USA
[5]Center for Computational Mathematics, Flatiron Institute, New York, NY 10010, USA
[6]Joint Department of Biomedical Engineering, University of North Carolina at Chapel Hill / North Carolina State University, Chapel Hill, NC 27599, USA
[7]Department of Bioengineering, Stanford University, Stanford, CA 94305, USA
[8]Howard Hughes Medical Institute, Stanford University, Stanford, CA 94305, USA
[9]Department of Electrical & Computer Engineering, Duke University, Durham, NC 27708, USA
[10]Duke School of Medicine, Department of Cell Biology, Duke University, Durham, NC 27708, USA
[11]Department of Biomedical Engineering, Duke University, Durham, NC 27708, USA

[‡]Current affiliation: Departments of Biomedical Engineering and Computational Medicine & Bioinformatics, University of Michigan, Ann Arbor, MI 48109, USA.

* Corresponding authors: adraelos@umich.edu, eva.naumann@duke.edu

[†]These authors jointly supervised this work.

Contents:

1. ***improv*** **installation and design**
2. ***improv*** **use cases and API**
3. **Supplementary Figures**

Supplementary Information

1. *improv* installation

System requirements. *improv* was designed to be as system-agnostic as possible. The core functionality is written for Python 3.10, but is compatible for Python 3.7 – 3.10, and requires the installation of a few other packages (such as numpy, pyarrow, and pyzmq). Installation of *improv* has been tested and confirmed on Linux (Ubuntu), Mac OS X, and Windows running Windows Subsystem for Linux (WSL).

Base installation. We provide complete installation instructions in the Readme file on the Github repository and through our documentation website: https://project-improv.github.io/improv/installation.html.

The easiest method for installation is through the Python Package Index PyPI:

```
pip install improv
```

Users can also use a local clone from Github to install the package, especially if they desire to edit or contribute to the codebase. Briefly, two calls are needed:

1. `python -m build` to build the package with all requirements,
2. `pip install -e .` to install the package inside the local directory.

After either installation method, *improv* can be executed on a particular user-configured setup with:

```
improv run experiment.yml
```

improv software design

Configuration in *improv* is streamlined and simple, requiring only that a user define (1) what processing steps (i.e., actors) are part of the pipeline, and (2) in what order they should be executed. The pipeline definition is lightweight: directed graphs are specified by text-based configuration files with two sections (**Supplementary Fig. 1**). In the first section, actors are specified by the names of their respective Python classes, with any additional information passed as keyword arguments to class constructors. In the second section, connections among actors are specified by listing the consumers for each actor's outputs. On startup, *improv* handles construction for each of these queues and links them with their respective actors. *improv* can handle both single and multiple output queues to accommodate arbitrary configurations.

Importantly, all instantiation, memory-sharing, execution flow, and logging are handled by *improv*, leaving the user free to focus on code within an actor. Furthermore, any Python code (or code executable in a Python environment) is acceptable within an actor: we do not supply a library of functions or require that a user follow particular implementation. Thus, almost anything a user wants to compute or run within an actor is available, including integration with other software tools.

Supplementary Fig. 1b illustrates this for a simple Processor actor. Here, users define the runStep method which is run in a rapid and infinite loop by *improv*. The method executes code to receive a data store ID (key) from the input queue and uses it to retrieve the datum (in this example, an array called self.frame of neural activity). It computes the frame average and places the result back into the data store. The keys to identify those results are then published in the supplied output queue self.q_out. In our example pipeline, these are retrieved for display by the visual actor (the graphical user interface). This actor can remain unaware of all other aspects of the experiment and awaits only the next piece of data to execute its computation.

improv only specifies the semantics of data pipelines, freeing users from the need to manage implementation details. This is facilitated by two key components: a shared, in-memory data store, discussed above, and a central controller program, dubbed Nexus. Once users have defined each processing step in the pipeline and the dependency relationships between them, Nexus is responsible for the actual orchestration of experiments. At runtime, Nexus instantiates each actor, configures information and execution flow, and handles both error signaling and user interactions. On startup, it takes in a list of desired actors and associated attributes from the configuration file, instantiates each class, and provides it access to the shared data store. *improv*'s user contract is thus both extensible and simple: users write custom Python classes for any new analyses they require, but they do not need to know the internals of classes implementing other pipeline steps.

Once the experimental pipeline is started, Nexus executes each class in a separate process and monitors its progress. Each process corresponding to an actor is kept alive continuously, waking as data become available in its input queue. In this way, the system leverages concurrency to overcome delays due to serial processing or input/output overhead. Communication is handled asynchronously, using a custom class combining Python's asyncio and multiprocessing libraries. As a result, the entire pipeline is robust to failures: no one actor or internal task can suspend the system, which would effectively cause the experiment to terminate. In addition, each object placed into the data store and every parameter change can be logged to disk, effectively creating a snapshot of the system state at each moment in the experiment. This ensures a robust audit trail, such that any data or associated analysis can be reproduced by later offline analyses.

2. API and Sample use cases

Graphical user interface. For our specific experimental integration of *improv* with a two-photon calcium imaging setup, we also constructed a simple graphical user interface (GUI) to provide user control and real-time updates of all images and analyses (**Supplementary Video 1**). For the paradigm described in the main text, we plot fluorescence or extracted spikes as a function of the last 500 frames (scrolling window) for both the population average and a user-selected neuron. Response profiles are plotting as circular tuning curves adjacent to those line plots. Raw images acquired from the setup are shown to the left, and the processed and analyzed frame is shown to the right. Neurons in the processed frame are colored by their tuned responses and can be selected via mouse click by a user to display its data above. On the right side we display the online results of the LNP model fit. The top plot shows the negative log-likelihood function being minimized as more frames are analyzed, and the bottom plot displays the inferred weight matrix of connections among the top 10 neurons with largest model weights. Again, by selecting a neuron in the center processed frame plot, the connections associated with that neuron are displayed as green lines.

Integration with other tools. While *improv* represents the only fully extensible tool dedicated in the online setting, there are a host of excellent tools available for offline analysis. As a step toward integration with these tools, we have implemented proof-of-concept interfaces between *improv* and both Suite2p (via its Python command-line interface) and ScanBox (via the MATLAB execution environment) (**Supplementary Fig. 5**). Other pipeline or workflow generating software packages have also tackled this problem of efficient pipelining, even specifically for the neuroscience community (Gorgolewski, 2011). Our system by contrast does not attempt to directly bundle collections of applications for a multi-use toolbox, but instead is a lightweight scaffolding approach that provides containers (actors) to accommodate most any application the user needs. This also ensures our code does not fall into maintenance traps, but rather easily enables updates or adding new functionality.

We additionally looked at integration across different programming languages. For instance, both Python and Julia have packages designed for interfacing with one another. Using the Python package PyJulia, we can compile and execute a piece of Julia code from within a Python program. Combining this with the Julia package PyCall to do the reverse, we can flexibly transfer data from *improv* to Julia for quick analysis (e.g., gradient descent of an LNP model) and transfer the results back into *improv* once again. Notably, this transfer between languages can be accomplished with time-

efficient no-copy wrappers if using NumPy arrays. For an example implementation using Julia see the julia branch at github.com/pearsonlab/improv.

Benchmarking

Our optimized system proved capable of preprocessing, analyzing, and visualizing results at rates faster than a simulated acquisition frequency of 30 Hz, which is roughly 6.7 MB/s for one test data set. Moreover, we show that even faster rates than this are possible. Whereas a serial implementation of the analysis exceeds the per cycle time budget of 33ms, *improv*'s inbuilt computational concurrency accomplishes all the same steps well within these time constraints, suggesting that this approach can also scale to volumetric light-sheet microscopy, e.g., for the entire zebrafish brain at 0.8 Hz for 41 planes (roughly 7 MB/s) (Ahrens, 2013). And while short periods of heightened preprocessing time can occur, any lag between acquisition and final analysis quickly dissipates. In fact, *improv* was able to process data continuously for more than twenty-four hours without crashing or accumulating more than a single frame of processing lag (**Supplementary Fig. 2**). Furthermore, concurrency allows *improv* to easily ignore “bad actors,” i.e., missing or slow frames that frequently lag or crash, and maintain the processing the pipeline. To illustrate system stability, we simulated an imaging experiment in which the ‘acquisition’ actor drops frames: rather than await missing frames that may never appear (suspending the experiment) or raising errors (halting the experiment), each actor continues its own execution loop, processing any future data when they become available. Importantly, once acquisition resumes, the entire system recovers automatically, without delays or increased processing time.

3. Supplementary Figures

Configuration file

```
actors:
  GUI:
    package: actors.visual_model
    class: DisplayVisual
    visual: Visual

  Acquirer:
    package: actors.acquire_tbif
    class: TbifAcquirer
    filename: data/zf_images.tbif
    framerate: 3.6

  Processor:
    package: actors.processor
    class: Processor
    init_filename: data/init_data.h5
    config_file: caiman_params.txt

  Visual:
    package: actors.visual_model
    class: CaimanVisual

  Analysis:
    package: actors.analysis_model
    class: ModelAnalysis


connections:
  Acquirer.q_out: [Processor.q_in, Visual.raw_frame_queue]
  Processor.q_out: [Analysis.q_in]
  Analysis.q_out: [Visual.q_in]
  Acquirer.stim_queue: [Analysis.input_stim_queue]
```

Actor definition

```python
from improv.actor import Actor
import numpy as np
import logging

logger = logging.getLogger(__name__)
logger.setLevel(logging.INFO)

class Processor(Actor):

    def setup(self):
        self.name = "Processor"
        self.frame = None
        self.avg_list = []
        self.frame_num = 1
        logger.info("Completed setup for Processor")

    def runStep(self):
        frame = None
        try:
            frame = self.q_in.get(timeout=0.001)

        except:
            logger.error("Could not get frame!")
            pass

        if frame is not None and self.frame_num is not None:
            self.done = False
            self.frame = self.client.getID(frame[0][0])
            avg = np.mean(self.frame[0])

            self.avg_list.append(avg)
            self.frame_num += 1
            self.q_out.put(avg)
```

Supplementary Fig. 1 | Code examples for *improv* configuration and execution

Left, Example configuration file defining a set of actors and a set of connections. Actors are defined by Python classes. Connections form a directed graph specified by listing the interactions among each actor node.

Right, Example class file defining an actor whose operation computes mean activity. The runStep method fetches data from the store based on keys passed from other actors; processes these data using custom Python code; and places the results into the data store.

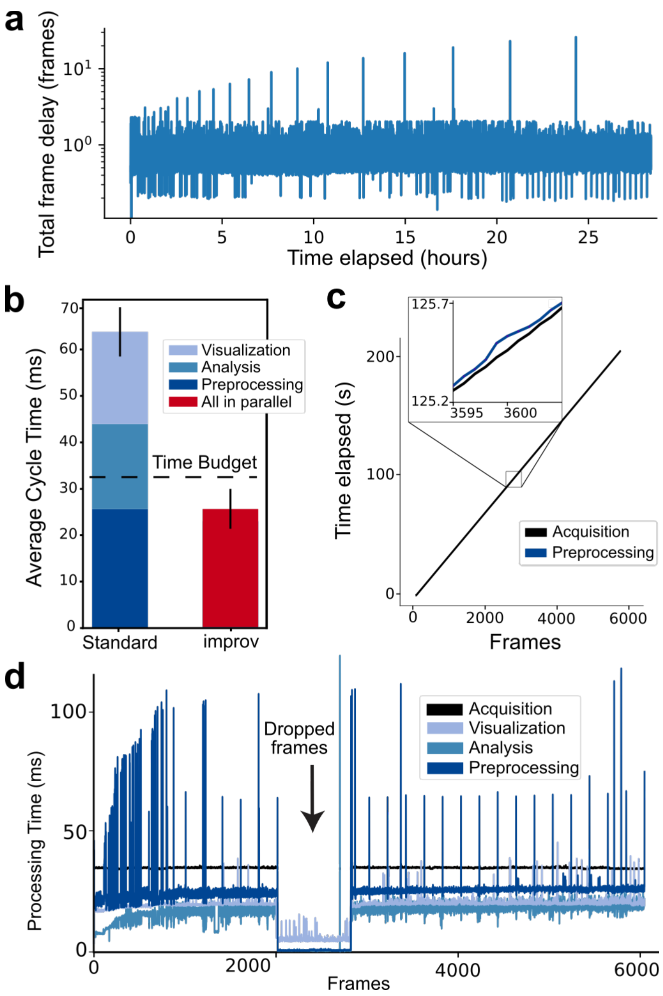

Supplementary Fig. 2 | Performance benchmarking for imaging experiments using prerecorded data

a, Total frame lag, the time between processing one frame and acquiring the next frame, shown over almost 30 hours running continuously. Spikes of larger frame delays observed periodically are due to rare computationally intensive functions to recalculate regions of interest in the Caiman Online actor. After such events and on average across the entire session there is less than 1 frame of total lag even after running for more than a day, demonstrating the robustness and feasibility of streaming data analysis.

b, *improv* parallelizes operations to reduce processing time. Real-time fluorescence image preprocessing, analysis, and visualization can be performed by improv in a total cycle time well below the time budget of 33 ms for data acquisition at 30 Hz. Executing each process in a standard offline processing sequence exceeds this benchmark, rendering online analysis impossible. Error bars show standard deviation for total average cycle times.

c, Timestamps for acquisition and preprocessing show stable processing time per frame. While some frames take longer than others to process (inset) the difference in data acquired and data processed does not accumulate.

d, Processing time per frame does not appreciably increase throughout the experiment. Even if frames are dropped or corrupted (here done manually to simulate such an event), actors can resume processing when data acquisition recovers.

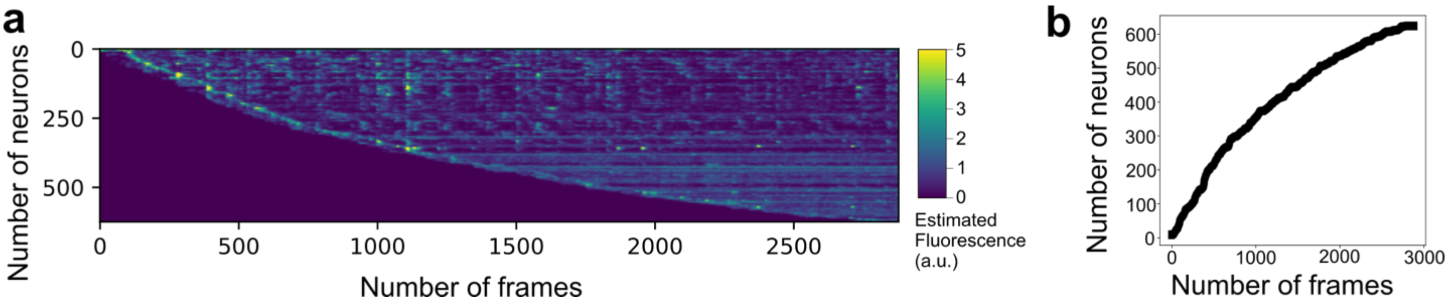

Supplementary Fig. 3 | CaImAn Online discovery of neurons across time

a, Colormap showing estimated fluorescence for each neuron as a function of the number of image frames observed. The dark blue region in the bottom left shows no initial activity for neurons identified later in the experiment. Neurons were identified and added throughout the experiment. More active neurons tend to be discovered earlier.

b, Total number of neurons identified as a function of the number of image frames observed. Fewer neurons are identified towards the end of the experiment compared to the beginning.

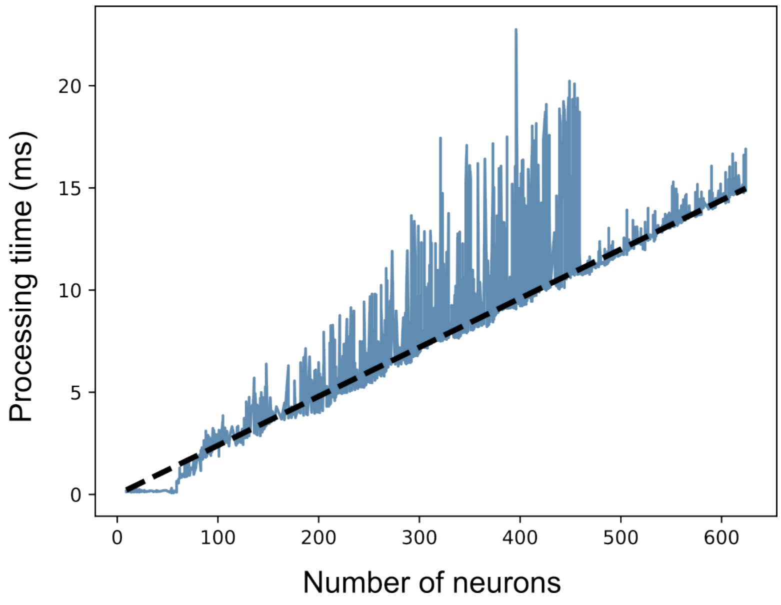

Supplementary Fig. 4 | Computation time as a function of number of neurons for LNP model

Processing time (in ms) plotted as a function of the number of neurons considered during LNP model fitting online for a single experiment (blue). Each datapoint is the amount of time it took to process that particular image. Recalculating the contours of neurons and adding new ones are the likely reasons for the increased processing times shown as sharper peaks. The dashed black line is a line of best fit (y = 0.024*x) and guide to the eye to show effective linear scaling for this experiment.

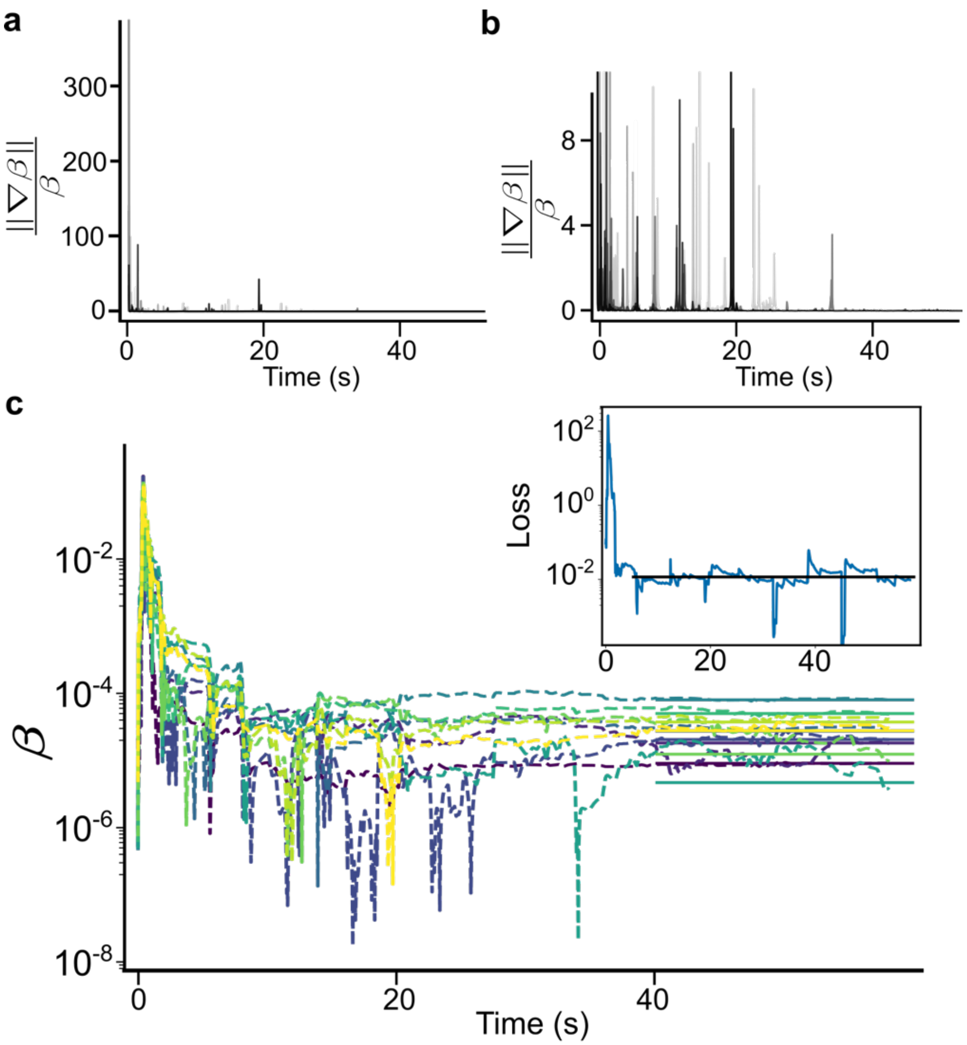

Supplementary Fig. 5 | Additional regression analysis of behavioral factors for Fig. 3

a, Scaled regression coefficients from **Fig. 3c** replotted using a linear scale on the y axis. Different grey lines correspond to different coefficients for each latent behavioral feature (10 total).

b, Same data from **a** with a zoomed-in y-axis scale to better show detail.

c, Convergence of online fit to an offline fit. Online values for beta are plotted as a function of time (dashed colored lines) and are shown to converge to similar beta values in the offline fit (solid-colored lines on the right). Different colors correspond to different coefficients for each latent behavioral feature (10 total). The inset shows the total loss (mean squared error plus L2 regularizer on the regression coefficients) for the online model fit as a function of time (blue line) compared to the offline model fit (black line).

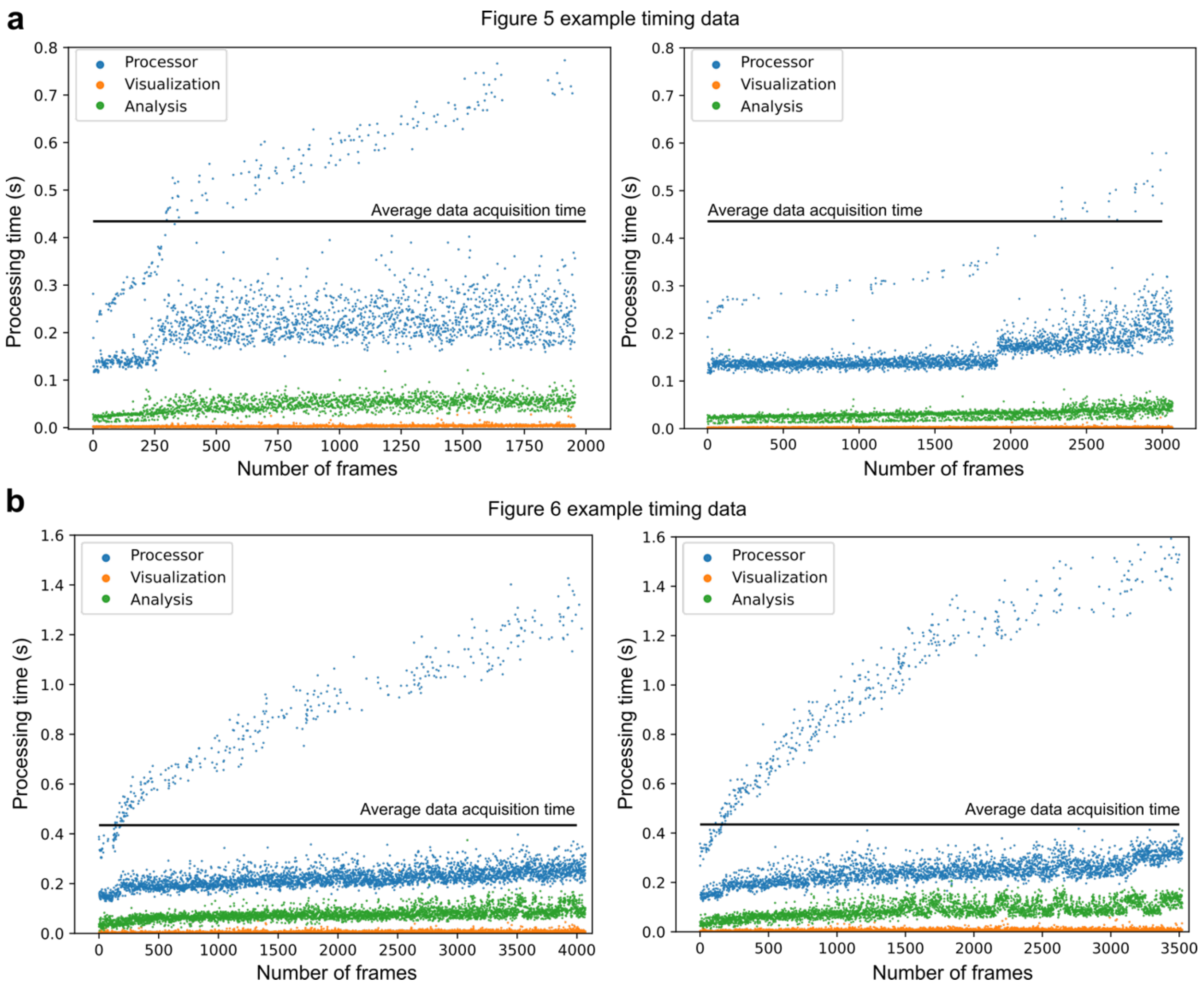

Supplementary Fig. 6 | Real-time time performance plots for online optimization in Figs. 5 & 6

a, Timing data for each major step in the experimental pipeline from two example experiments associated with the Bayesian optimization work in **Fig. 5**. Analysis here denotes the Bayesian optimization and neural tuning curve analyses. The abrupt step up in processing time for the Processor (Caiman Online) actor are when the number of neurons tracked increased above a threshold and the array containing activity estimates was reallocated with more memory. Blue dots above the average data acquisition line (black) are rarer times when the Processor actor recomputed the shapes of tracked neurons. Overall lag after these times quickly dropped back to zero and the experiment continued without interruption.

b, Timing data for each major step in the experimental pipeline from two example experiments associated with the photostimulation optimization work in **Fig. 6**. Analysis here denotes the neural tuning curve and photostimulation target selection model.

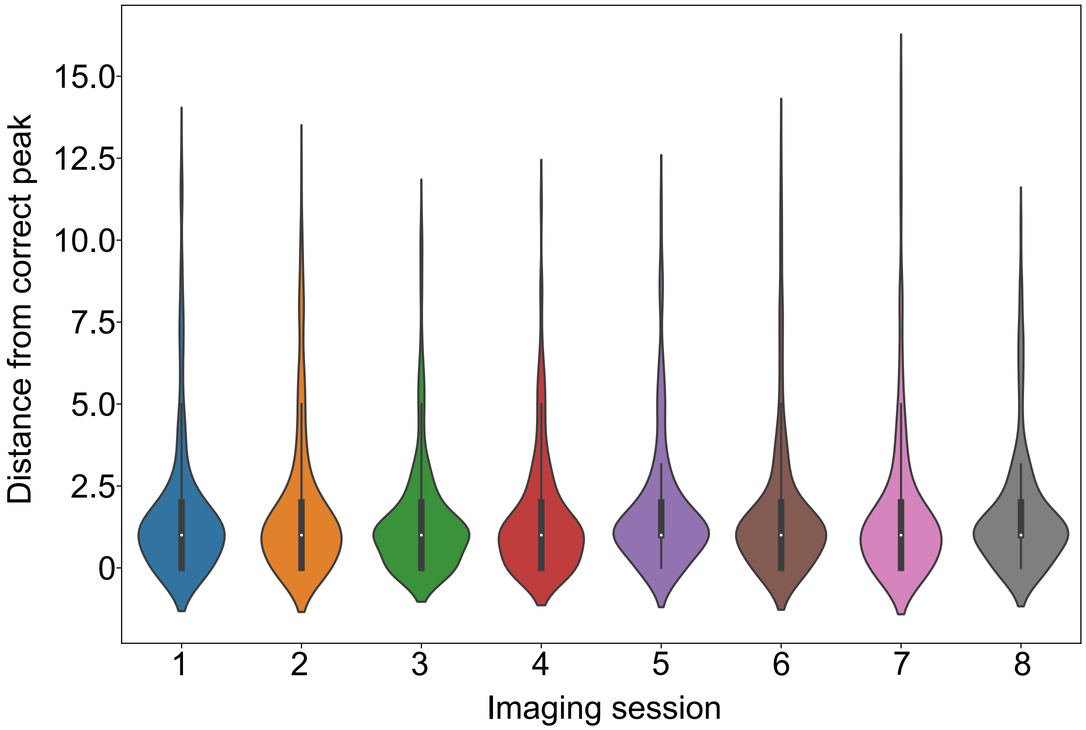

Supplementary Fig. 7 | Comparing offline versus online peak neural activity finding

Violin plot showing the distances from the online identified peak neural activity to the 'correct' peak, defined as the peak obtained from later offline fitting, for each optimized neuron across 8 different imaging sessions (roughly 300 neurons per session). In most cases, the peak identified online was less than 2 stimuli away: 15 degrees in each eye or 30 degrees in a single eye.

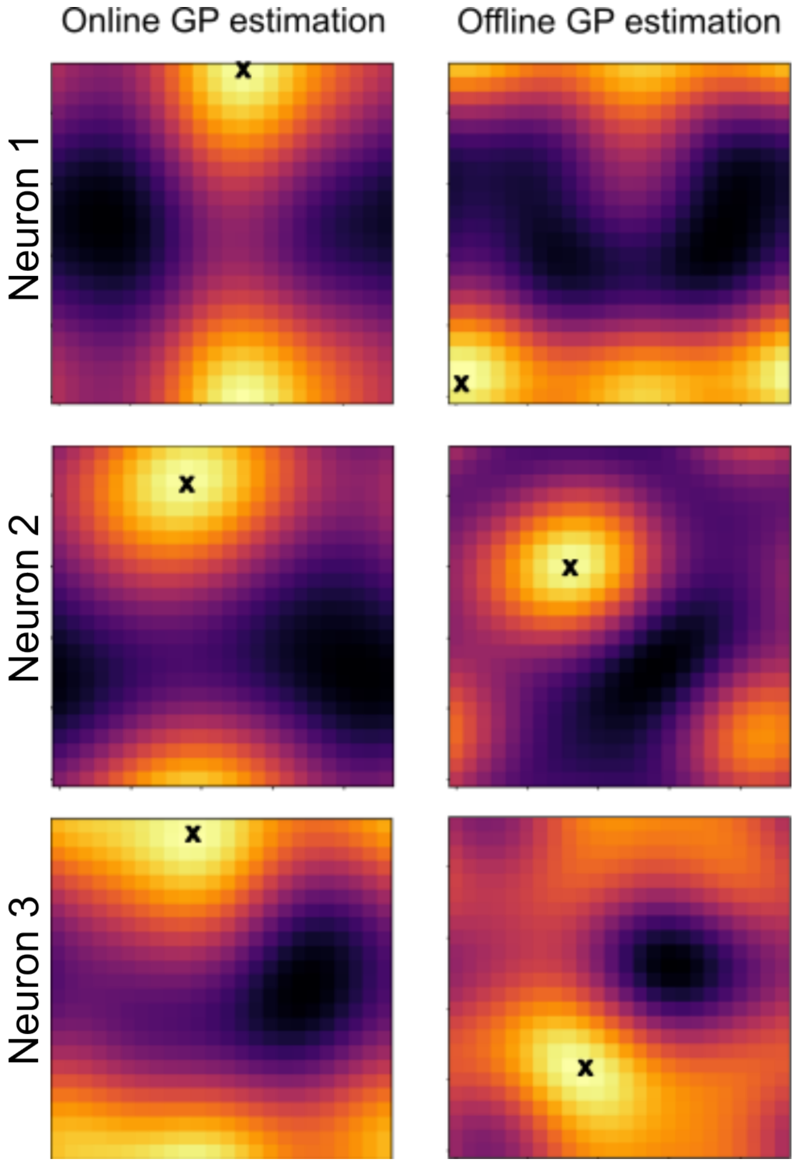

Supplementary Fig. 8 | Failure modes for online GP estimation during response peak finding

The estimated neural responses to binocular visual stimuli during the online optimization routine (*left*) and using an offline fit to all data collected (*right*) for 3 example neurons with poor agreement between the conditions. In each case, a black 'X' denotes the identified peak location. For these, multimodal or warped peak structures were found in the offline fits, violating our model assumption of a single 2d Gaussian peak. Consequently, their peak locations were not successfully identified.

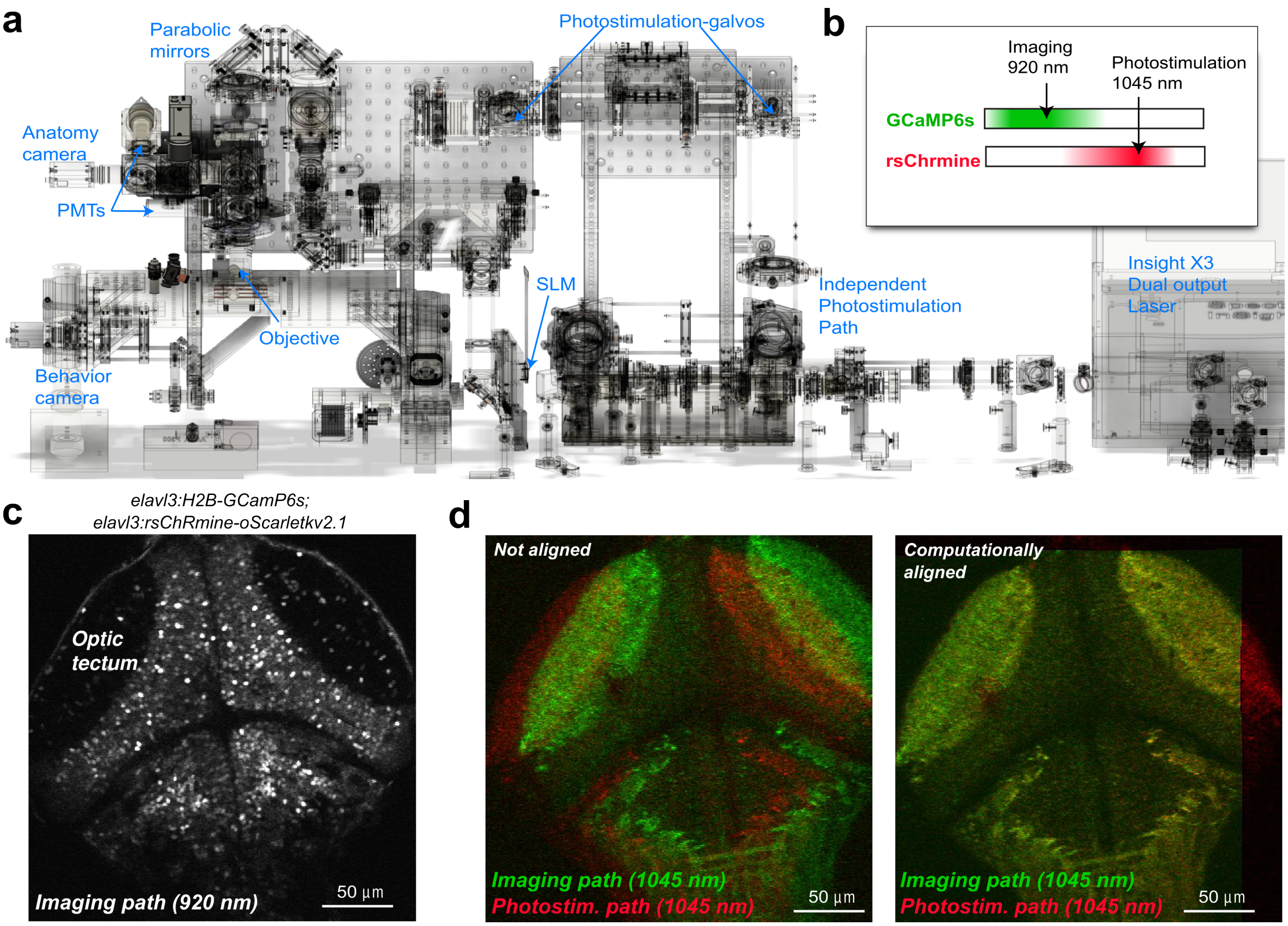

Supplementary Fig. 9 | Alignment of calcium imaging and optogenetic photostimulation targets

a, Schematic of the design of the two-photon microscope for simultaneous calcium imaging and photostimulation. A two-photon laser (InsightX3) generates 920 nm excitation and 1045 nm simultaneously. Two independent optical paths allow for laser scanning imaging with both wavelengths at the same focal point in z at the objective. The photostimulation optical path also contains galvo mirrors and spatial light modulator for 3D precision generation of photoactivating spiral patterns.

b, Diagram illustrating the spectral separation of imaging GCaMP6s with 920 nm and photostimulated of rsChRmine with 1045 nm, minimizing photoactivation of neurons while measuring neural activity by exciting GCaMP6s.

c, Example maximum intensity image obtained from the imaging path at 920 nm showing the optic tectum showing nuclear-located GCaMP6s activated (brighter neurons).

d, Demonstration of computational alignment process using images obtained via the imaging and photostimulation paths. Both images were obtained with 1045 nm excitation. Images show images of both channels before and after computational alignment. The same computational alignment was used to calculate accurate photostimulation target locations.

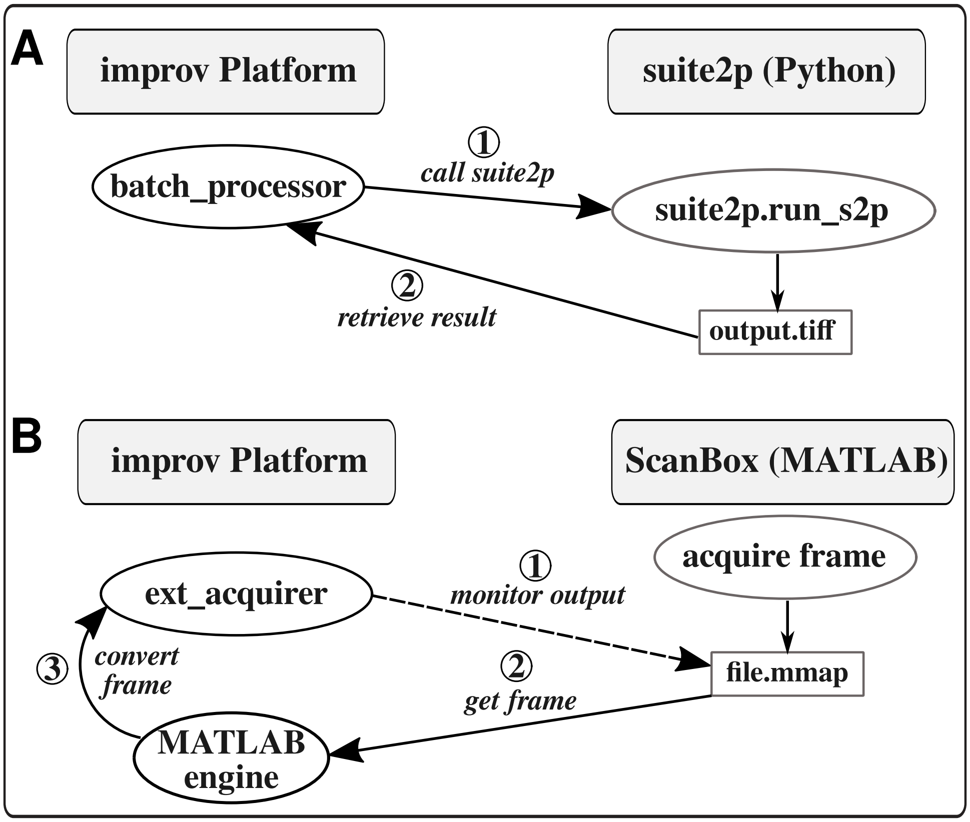

Supplementary Fig. 10 | Integration with other acquisition and analysis tools

a, *improv* can use suite2p for analysis by using our own 'batch_processor' actor that calls suite2p via its Python interface. Outputs from suite2p are saved to disk and read back into improv's data store for further processing.

b, *improv* can be used to analyze data acquired by ScanBox via our 'ext_acquirer' actor to monitor the memory-mapped (mmap) file used by ScanBox to write data to disk. When needed, it can access these data by calling MATLAB from Python via the MATLAB engine.
