# Supplementary material for: A software platform for real-time and adaptive neuroscience experiments
Source: Nat Commun. 2025 Nov 11;16:9909. doi: 10.1038/s41467-025-64856-3 (PMC12606217; doi:10.1038/s41467-025-64856-3)
Supplement: Supplementary file 2 — Description of Additional Supplementary Files [file 41467_2025_64856_MOESM2_ESM.pdf]

Description of Additional Supplementary Files:

Supplementary Video 1

A screen recording of the improv GUI shown in Fig. 2 illustrates streaming analysis of directional tuning computed from calcium imaging via two-photon microscopy. The color of each neuron encodes continuously updated directional preferences across the length of the recording, see color wheel in Fig. 2d. Green lines between neurons predict connectivity across the brain. Similar GUIs were used for in vivo experiments shown in Fig. 5 and Fig. 6.

Supplementary Video 2

Online regression coefficients overlaid onto the original behavioral video of the mouse's face and paws for the experiment described in Fig. 3. Color intensity corresponds to the instantaneous magnitude of the coefficients across the image.

Supplementary Video 3

The video illustrates live data acquisition during Bayesian optimization for each for every new visual stimulus in Fig. 5. Left, Calcium fluorescence traces for each stimulus window. Center, the Gaussian process tuning curve. Right, associated uncertainty. The overlaid dots denote the sampled location (visual stimulus combination) with the color corresponding to the measured neural activity in response to that stimulus.

Supplementary Video 4

Example two-photon calcium fluorescence recording during visual stimulation and photostimulation shown in Fig. 6. The top left symbols indicate the currently displayed visual stimulus or the time of photostimulation (red dot). Both visual stimuli or photostimulation events cause increases in fluorescence in various neurons across the field of view.

Supplementary Video 5

A screen recording demonstrating the text interface supplied with improv and basic usage. An experiment is loaded from a .yaml file, initialized with the setup command, then run,

stopped, and quit using typed commands. Log messages are displayed in the top portion of the window, with user input accepted in the below portion.

Supplementary Data 1: These data contain the data shown in Figure 2f.

Supplementary Data 2: These data contain the data shown in Figure 3b.

Supplementary Data 3: These data contain the data shown in Figure 3c.

Supplementary Data 4: These data contain the data shown in Figure 5e.
