# Supplementary material for: A software platform for real-time and adaptive neuroscience experiments
Source: Nat Commun. 2025 Nov 11;16:9909. doi: 10.1038/s41467-025-64856-3 (PMC12606217; doi:10.1038/s41467-025-64856-3)
Supplement: Supplementary file 12 — Reporting Summary [file 41467_2025_64856_MOESM12_ESM.pdf]

nature portfolio

Corresponding author(s): Naumann, E.A / Draelos A.

Last updated by author(s): 2025/09/05

Reporting Summary

Nature Portfolio wishes to improve the reproducibility of the work that we publish. This form provides structure for consistency and transparency in reporting. For further information on Nature Portfolio policies, see our Editorial Policies and the Editorial Policy Checklist.

Statistics

For all statistical analyses, confirm that the following items are present in the figure legend, table legend, main text, or Methods section.

n/a	Confirmed	
☒	☐	The exact sample size (*n*) for each experimental group/condition, given as a discrete number and unit of measurement
☒	☐	A statement on whether measurements were taken from distinct samples or whether the same sample was measured repeatedly
☒	☐	The statistical test(s) used AND whether they are one- or two-sided<br>*Only common tests should be described solely by name; describe more complex techniques in the Methods section.*
☒	☐	A description of all covariates tested
☒	☐	A description of any assumptions or corrections, such as tests of normality and adjustment for multiple comparisons
☒	☐	A full description of the statistical parameters including central tendency (e.g. means) or other basic estimates (e.g. regression coefficient) AND variation (e.g. standard deviation) or associated estimates of uncertainty (e.g. confidence intervals)
☒	☐	For null hypothesis testing, the test statistic (e.g. *F*, *t*, *r*) with confidence intervals, effect sizes, degrees of freedom and *P* value noted<br>*Give P values as exact values whenever suitable.*
☒	☐	For Bayesian analysis, information on the choice of priors and Markov chain Monte Carlo settings
☒	☐	For hierarchical and complex designs, identification of the appropriate level for tests and full reporting of outcomes
☒	☐	Estimates of effect sizes (e.g. Cohen's *d*, Pearson's *r*), indicating how they were calculated

Our web collection on statistics for biologists contains articles on many of the points above.

Software and code

Policy information about availability of computer code

Data collection	Data collection was guided by our software platform, improv. This software package can be downloaded from github.com/project-improv/improv or automatically installed via the Python Package Index pypi.org/project/improv. Additional integration code between improv and the microscope LABView system is available at a forked repository: https://github.com/Naumann-Lab/improv.<br><br>To generate visual stimuli, we used our package pandastim, available at www.github.com/Naumann-Lab/pandastim.
Data analysis	Data analysis was conducted by our software platform, improv. This software package can be downloaded from github.com/project-improv/improv or automatically installed via the Python Package Index pypi.org/project/improv. The improv software package can be downloaded from github.com/project-improv/improv or the Python Package Index pypi.org/project/improv. It can also be found at DOI:10.5281/zenodo.17079045 and https://zenodo.org/records/17079045.

For manuscripts utilizing custom algorithms or software that are central to the research but not yet described in published literature, software must be made available to editors and reviewers. We strongly encourage code deposition in a community repository (e.g. GitHub). See the Nature Portfolio guidelines for submitting code & software for further information.

Data

Policy information about availability of data

All manuscripts must include a data availability statement. This statement should provide the following information, where applicable:

- Accession codes, unique identifiers, or web links for publicly available datasets
- A description of any restrictions on data availability
- For clinical datasets or third party data, please ensure that the statement adheres to our policy

All new data generated for this paper is made freely available for research purposes and hosted on DANDI at https://dandiarchive.org/dandiset/001569.
We also used the following publicly accessible datasets: https://dx.doi.org/10.14224/1.38599, https://dx.doi.org/10.5281/zenodo.3854034

Research involving human participants, their data, or biological material

Policy information about studies with human participants or human data. See also policy information about sex, gender (identity/presentation), and sexual orientation and race, ethnicity and racism.

Reporting on sex and gender	N/A
Reporting on race, ethnicity, or other socially relevant groupings	N/A
Population characteristics	N/A
Recruitment	N/A
Ethics oversight	N/A

Note that full information on the approval of the study protocol must also be provided in the manuscript.

Field-specific reporting

Please select the one below that is the best fit for your research. If you are not sure, read the appropriate sections before making your selection.

☒ Life sciences ☐ Behavioural & social sciences ☐ Ecological, evolutionary & environmental sciences

For a reference copy of the document with all sections, see nature.com/documents/nr-reporting-summary-flat.pdf

Life sciences study design

All studies must disclose on these points even when the disclosure is negative.

Sample size	Our sample sizes for zebrafish imaging data are similar to those reported in previous publications (Naumann et al., 2016, Kramer et al., 2019). These include approximately N=3-6 animals for two photon imaging in zebrafish. For existing publicly available datasets with data from mouse and monkey, we used N=1 animal .No statistical methods were used to pre-determine the number of subjects in this study.
Data exclusions	For zebrafish studies, no animals were excluded from analyses, if their health (heart beat, blood flow, spontaneous neural activity) indicated good health. For photostimulation experiments (figure 6), only animals that showed consistent neural activity changes via calcium imaging when photostimulated were included in further analysis.
Replication	All zebrafish recordings were performed with multiple animals per group (minimum of N=3, n = 300 neurons) and yielded consistent, reproducible neural activation patterns. To confirm that data were reproducible for analysis in Figure 1, we ran calcium imaging analysis over 10 times, each time confirming similar numbers of direction selective neurons. For Figure 5, we replicated visual peak tuning results across 15 imaging sessions per fish. For Figure 6, we replicated results across 3 fish, reproducing the findings that most visually responsive neurons also can be optogenetically photostimulated.
Randomization	For all new zebrafish studies, individuals of a specific genotype were selected randomly for imaging and photostimulation in the pretectum and optic tectum. All visual stimuli were presented in pseudo random sequence unless adaptively selected by our Bayesian model.
Blinding	For zebrafish studies, blinding was not feasible because the same experimenters chose animals, image planes and software parameters. To minimize bias, we employed standardized and automated procedures for data collection, parameter selection and analysis where applicable.

Reporting for specific materials, systems and methods

We require information from authors about some types of materials, experimental systems and methods used in many studies. Here, indicate whether each material, system or method listed is relevant to your study. If you are not sure if a list item applies to your research, read the appropriate section before selecting a response.

Materials & experimental systems

n/a	Involved in the study
☒	☐ Antibodies
☒	☐ Eukaryotic cell lines
☒	☐ Palaeontology and archaeology
☐	☒ Animals and other organisms
☒	☐ Clinical data
☒	☐ Dual use research of concern
☒	☐ Plants

Methods

n/a	Involved in the study
☒	☐ ChIP-seq
☒	☐ Flow cytometry
☒	☐ MRI-based neuroimaging

Animals and other research organisms

Policy information about studies involving animals; ARRIVE guidelines recommended for reporting animal research, and Sex and Gender in Research

Laboratory animals	For new live experiments in Figure 5 and 6, experimental subjects were zebrafish 5-10 days old (Danio rerio) in Casper background. For Figure 5, we used Tg(elavl3:H2B-GCaMP6s) , for Figure 6 we used double transgenic fish Tg(elavl3:H2B-GCaMP6s); Tg (elavl3:rsChRmine-oScarlet-Kv2.1 generated in this study.
Wild animals	N/A
Reporting on sex	The zebrafish sex is not established before approximately 21 days of age. Since all research in this study was performed exclusively in the larval stages (less than 10 days post fertilization), sex is not a relevant biological variable in this study.
Field-collected samples	N/A
Ethics oversight	Duke University's standing committee on the use of animals in research and training (DUKE UNIVERISTY IACUC) protocol #: A058-24-03.

Note that full information on the approval of the study protocol must also be provided in the manuscript.

Plants

Seed stocks	N/A
Novel plant genotypes	N/A
Authentication	N/A
